# Testing the effects of brief intervention in primary care for problem drug use in a randomized controlled trial: rationale, design, and methods

**DOI:** 10.1186/1940-0640-7-27

**Published:** 2012-12-14

**Authors:** Antoinette Krupski, Jutta M Joesch, Chris Dunn, Dennis Donovan, Kristin Bumgardner, Sarah Peregrine Lord, Richard Ries, Peter Roy-Byrne

**Affiliations:** 1 Department of Psychiatry and Behavioral Sciences, University of Washington at Harborview Medical Center, Seattle, WA, USA; 2 Alcohol and Drug Abuse Institute, University of Washington, Seattle, WA, USA

**Keywords:** Problem drug use, Screening, Brief Intervention and Referral to Treatment (SBIRT), Motivational Interviewing (MI), Addiction Severity Index (ASI), Safety net, Public health benefit, Cost effectiveness

## Abstract

**Background:**

A substantial body of research has established the effectiveness of brief interventions for problem *alcohol use.* Following these studies, national dissemination projects of screening, brief intervention (BI), and referral to treatment (SBIRT) for alcohol *and drugs* have been implemented on a widespread scale in multiple states despite little existing evidence for the impact of BI on drug use for non-treatment seekers. This article describes the design of a study testing the impact of SBIRT on individuals with drug problems, its contributions to the existing literature, and its potential to inform drug policy.

**Methods/design:**

The study is a randomized controlled trial of an SBIRT intervention carried out in a primary care setting within a safety net system of care. Approximately 1,000 individuals presenting for scheduled medical care at one of seven designated primary care clinics who endorse problematic drug use when screened are randomized in a 1:1 ratio to BI versus enhanced care as usual (ECAU). Individuals in both groups are reassessed at 3, 6, 9, and 12 months after baseline. Self-reported drug use and other psychosocial measures collected at each data point are supplemented by urine analysis and public health-related data from administrative databases.

**Discussion:**

This study will contribute to the existing literature by providing evidence for the impact of BI on problem drug use based on a broad range of measures including self-reported drug use, urine analysis, admission to drug abuse treatment, and changes in utilization and costs of health care services, arrests, and death with the intent of informing policy and program planning for problem drug use at the local, state, and national levels.

**Trial registration:**

ClinicalTrials.gov NCT00877331

## Background

A substantial body of research has established the efficacy and effectiveness of brief (one to two sessions) interventions (BI) for excessive or hazardous alcohol use in patients seen in medical settings, both primary care and emergency department (ED) [1-3]. Following these studies, national dissemination projects of screening, brief intervention, and referral to treatment (SBIRT) for alcohol *and drugs* have been implemented on a widespread scale in multiple states [4,5]. Some have argued that this rapid progression of BI from efficacy to effectiveness to dissemination for drugs other than alcohol has outstripped its evidence base [6]. For example, there have been few randomized controlled trials of BI for drug abuse in the general medical setting for non-treatment seekers [7,8], leading the US Preventative Services Task Force to conclude that the utility of BI in medical settings as an opportunistic intervention for drug abuse remains unclear [9]. Thus, from a policy perspective, an important but still unanswered question is whether BI reduces drug use.

According to a recent epidemiological study, a disproportionate number of individuals with drug abuse or dependence are from lower socioeconomic strata [10], where access to specialized substance abuse treatment is difficult and, as a result, exacerbates the motivational challenges and follow-through that BI targets. Such individuals, who are often uninsured or on Medicaid, face daunting and diverse barriers to health care access and are usually served in public sector safety net medical settings (hospitals and community health clinics). Few of these individuals seek specialist chemical dependency treatment [11]. The related costs of untreated drug abuse are substantial [12-14]. Updated data on such costs and public health-related adverse effects are needed by policymakers to create the necessary traction to drive policy changes to improve provision of drug treatment. In this regard, ambulatory primary care is an important setting to test the effectiveness of BI on drug abuse since, were it effective, modest effects for a large number of patients would yield a sizable public health benefit [15].

Accordingly, this study was designed to evaluate the impact of a BI on patients with problem drug use in a primary care setting within a safety net system of care. It is intended to serve as a policy-relevant randomized controlled trial with broad external validity. It builds upon the significant knowledge base acquired in the study of brief interventions in medical settings for alcohol abuse including a well-documented intervention utilizing motivational interviewing (MI) [16-18] and a growing literature that focuses on public health outcomes of BI [19,20]. It is unusual in its focus on providing BIs to individuals with evidence of *drug dependence and abuse* in primary care. As such, its design and implementation has required consideration of a number of factors that, prior to this point in time, have not been relevant to the study of BIs. The purpose of this paper is to describe the design as well as the rationale for key decisions made in this study with the intent of informing future studies with a focus on BI in drug abuse.

## Methods and design

### Specific aims

The primary focus of the study is to evaluate the impact of a BI on patients with problem drug use and abuse seen in a primary care clinic at a large safety net hospital by comparing outcomes among patients randomly assigned to one of two conditions: (i) BI, consisting of one encounter during a routine primary care medical visit with a brief follow-up phone booster and referral to treatment when indicated; and (ii) enhanced care as usual (ECAU) consisting of problem notification and referral during a routine primary care medical visit. Specific aims are to: (1) Examine whether BI is effective compared to ECAU at reducing drug use and improving psychiatric and select psychosocial outcomes in individuals with a spectrum of problem drug use; (2) Test whether interventionist fidelity to the BI model is associated with better outcomes; (3) Estimate the impact of the BI on several public health outcomes that are directly related to the hazardous effects of illicit drug use including the use of acute health care services, involvement in the criminal justice system, HIV risk behavior, and mortality; and (4) Estimate the costs of the intervention, potential cost offsets, and its incremental cost-effectiveness from the payer perspective based on health care service use and drug use frequency.

### Study design

**Figure 1:**
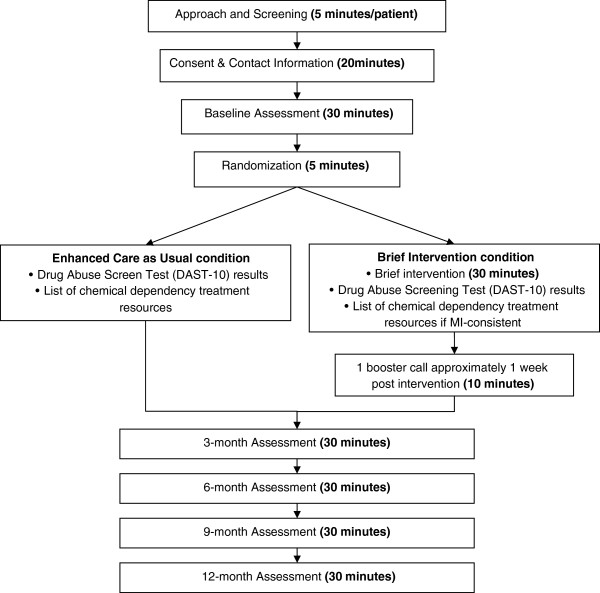
Study design.

This study is a two-group randomized prospective trial with blinded assessments (See Figure 1), approved by the University of Washington Institutional Review Board (Protocol #34892). Written informed consent is obtained from patients for study participation including permission to use unidentified information collected from them in study reports. Approximately 1,000 individuals presenting for scheduled medical care at one of seven designated primary care clinics who endorse problematic drug use when screened during that visit and then randomized in a 1:1 ratio to BI versus ECAU using a stratified permuted block randomization procedure. The purpose of the stratification is to balance random assignment by three factors known to be related to substance abuse outcomes including severity of drug problem [21], presence of a co-occurring mental disorder [22], and readiness to change [23].

Individuals randomized to the BI group receive screening, a single-encounter BI with referral to treatment when indicated, and a single follow-up phone contact approximately one week later. Individuals assigned to ECAU receive screening, a handout with the results of their drug screen, the risk associated with their drug use, and a list of referrals to chemical dependency treatment. Individuals in both groups are re-assessed at 3, 6, 9, and 12 months after baseline. All assessments are carried out in person wherever possible, with phone interview as a back-up option. Persons conducting follow-up assessments are blinded to the participant’s intervention versus control group assignment.

### Two-arm design: BI versus usual care

A two-arm design was adopted in order to assess the effects of the BI over and above usual care. However, the clinic sites where recruitment takes place for this study offer patients more options than may be available in other safety net medical settings across the country. This fact coupled with the ethical need to provide referral following screening of dependent patients led to defining the control condition as ECAU. This condition provides a test of whether BI is better than a less expensive but relatively high quality usual-care option, as opposed to being better than no intervention at all (neither systematic screening nor follow-up of screen-positive patients) and, as such, represents a rigorous comparison.

### Influence of the SAMHSA SBIRT dissemination program

Washington State received an SBIRT grant from the Substance Abuse and Mental Health Services Administration (SAMHSA) in 2003 that placed chemical dependency professionals trained in BI and associated MI skills into the emergency departments (EDs) of nine hospitals across the state (“WASBIRT”) [20]. The site of the current study is one of these hospitals. In order to build upon the strengths of these WASBIRT ED services and to maintain consistency with them, the current study is set up to mirror the WASBIRT design to the extent that is both reasonable and possible. This includes using the Drug Abuse Screening Test (DAST-10) to estimate drug use severity, using physician extenders to deliver the intervention, and using a single 30–45 minute BI session.

When SAMHSA funding ended in 2008, local resources were made available to sustain SBIRT services in the ED at the site of the current study. This created two potential confounds. One, enrolled patients may receive a previous BI, and two, they may receive a subsequent BI. To control for these potential confounds, we use administrative data. Subsequent exposure is used as an outcome because those receiving a BI in the current study might be more open to receive an additional one, suggesting a useful metric for assessing intervention value.

### Defining the population

As stated earlier, the population of interest is individuals seeking primary care treatment in a safety net medical center setting. Screening procedures are designed to identify individuals with problem drug use along a continuum of severity. Study inclusion criteria include adults age 18 and over; having used an illegal drug or used a prescription medication for nonmedical reasons at least once in the past three months [24] (equivalent to a DAST-10 score greater than 0 [25,26]); currently receiving care in the primary care clinic and planning to continue such care for the next year; English speaking and able to read and understand screening and assessment forms (6^th^ grade literacy); and having a phone, easy access to a phone with voicemail, or access to e-mail in order to facilitate scheduling.

To enhance external validity there are few baseline exclusion criteria. They include having attended formal treatment for substance abuse (excluding self-help groups such as Alcoholics Anonymous) in the past month; life-threatening medical illness (potential mortality in next year); severe cognitive impairment or active psychosis interfering with cognitive ability to process the BI (based on performance during consent and assessment); or active high risk suicidality [27].

There are two reasons for not excluding patients with the most severe drug problems from this trial. First, although some studies of BI for alcohol have found that patients with alcohol dependence responded less well to a BI than patients with less severe abuse or hazardous drinking, Field recently found that patients with alcohol dependence responded better to a BI than those with less severe problems [28] and Cobain found hospital-based BI effects to be independent of baseline dependence or medical comorbidity [29]. Second, even if a BI were inadequate as a stand-alone treatment for safety net patients with severe drug dependence, BI may prove to be a valuable referral mechanism for getting these patients to more intensive chemical dependency treatment, as it did in WASBIRT [30]. For these reasons, we include individuals across the full range of drug use and abuse in the present study. To balance drug use severity across BI and control groups, we use the DAST-10 score as a stratifying variable.

### Rationale for selecting primary care versus ED setting

Providing a BI in the ED setting has a number of advantages. For example, the ED is where individuals with substance-related injuries and acute medical problems present for urgent care. This setting maximizes the chance for a “teachable moment” and has more “high risk” individuals with a greater potential for high cost medical care use. Although the prevalence of illicit drug use is greater in the ED [31], primary care sees a larger number of individuals on an annual basis and has a service system more amenable to ancillary behavioral treatments because of its chronic illness focus. Furthermore, risk reduction in this setting can potentially prevent higher cost ED and hospital visits since patients in safety net medical settings are less likely to utilize primary care in a ‘health maintenance’ fashion and more likely to schedule primary care appointments on an ad hoc basis when they have emergent medical problems. In individuals with hazardous drug use, some of these problems are substance-related, creating ‘teachable moments’ like those seen in patients seeking care in the ED. Finally, a host of prior studies have shown that integration of mental health into primary care is clinically and cost effective [32]. Integration of interventions for substance abuse into primary care could have similar advantages, especially since substance-related medical problems could be addressed at the same time [33-35].

### Assessing public-health benefits of the intervention

As described earlier, providing SBIRT services to a sizable proportion of individuals with hazardous substance abuse in the ambulatory primary care setting has the potential to result in a sizable public health benefit [15]. That is, the public health benefit, or population attributable benefit, is far greater if one affects many people in a small way than fewer people in a larger way. As such, it is important to examine this benefit, especially at a time when providers, health care administrators, and policy makers are being increasingly required to rein in health care costs that are spiraling upward while resources for public health care are diminishing [36]. Quantifying the impact of brief interventions on the cost of health care, public safety, and mortality represent critical information needed to inform policy and program planning at the local, state, and national levels. The current study was designed to respond to this need by making preliminary estimates of the public health-related benefits of the intervention including changes in use of inpatient, emergency department, and outpatient medical services; arrests; HIV infection; mortality; and costs through the use of administrative data.

### Keeping the assessment brief but accurate

To enhance external validity, we are limiting the intensity and frequency of assessment to bridge the distance between a traditionally rich and extensive efficacy study assessment battery and what might more routinely be performed as part of quality assurance when an SBIRT program is implemented. This decision also enhances internal validity because prolonged assessor contact with significant focus on substance-related measures (in the usual care arm) may have an unintended therapeutic effect as noted by other experts [15,37-41]. That is, the more elaborate assessment of the typical efficacy study would likely last longer than the actual BI itself and might confound the comparison condition of ECAU. As such, we are limiting assessment to 30 minutes, a duration which has been shown to not have an intervention effect in recent analysis [42]. At the same time, we are supplementing our assessment with administrative data that will allow us to verify medical services received and whether individuals had participated in substance abuse treatment recently or had received a BI outside of this study. Our access to medical records will also help us determine whether individuals with urine screens positive for opiates might be receiving prescription opiates for related painful medical conditions as well as whether there is documentation of excess use, withdrawal symptoms, requests for early prescriptions, or visits to the ED to obtain more opiates that can be used to recode self-reported opiate use gathered during assessment.

### Selecting study sites

The present study is sited in primary care clinics affiliated with a large urban safety net medical center. One advantage of this siting is that patients receive all of their urgent and emergent medical care at the medical center, making it possible to track medical utilization over time. Practical considerations require that participating clinics serve a high volume of patients and, additionally, have evidence that a reasonable proportion of patients (say, 25% or more) are likely to have problem drug use. Insuring sufficient numbers of women in the sample to maximize generalizability is another important consideration. Initially it was estimated that all study participants could be recruited from the pool of patients who sought treatment at one of four primary care clinics. After the first year, however, we were unable to recruit the numbers of patients suggested by our original estimates, which required us to expand to three additional medical clinics. Furthermore, the economic downturn prompted some clinic infrastructure and service delivery changes at the safety net medical center which operates these clinics, creating additional unanticipated challenges, for example, waiting lists for new patients and deciding to move all female primary care patients without specific obstetric and gynecologic needs to other primary care clinics.

### Specific nature of the intervention

The study employed a total of 17 interventionists. We decided to have social workers who were already employed in each of the clinics conduct the BI as they were more available and less costly than primary care physicians. We began with eight social workers and, as the study evolved, required additional interventionists. We recruited three additional social workers who were working in the clinics, two social workers not working in the clinics, two clinical psychology doctoral students, and two bachelor-level individuals with experience conducting structured clinical interviews.

The intervention consists of a single-encounter BI followed by an attempted single phone booster contact one week later. The BI is delivered using an MI approach following a rich history of successful MI-based SBIRT interventions in primary care and trauma center trials for hazardous alcohol use over the last 15 years [18,43-45]. Also, a central consideration in choosing MI for delivery of the intervention was its documented larger effect sizes with ethnic minorities [46]. With every patient, interventionists perform standard elements or “clinical tasks”. The clinical tasks of the counseling session include orienting patients to the purpose of the session, telling patients their screening results, discussing options for change, and giving patients information about links between drug abuse and medical conditions. Interventionists perform these tasks using the highest quality MI skills of which they are capable. Two examples may serve to clarify how one might perform the clinical tasks using an MI style. One example is, when giving screening feedback, the interventionist uses a standard explanation of the DAST-10 score to help the patient understand the meaning of their score while, at the same time, using specific MI skills to handle any arguing or objections from the patient that may arise upon hearing their score. These skills might include reflecting the patient’s surprise or gleaning from the patient’s arguments apparent personal strengths such as having a genuine concern about whether drugs are hurting him/her. A second example of integrating MI illustrates our effort to enhance the potential “teachable moment” of the protocol. This task involves focusing the interview on specific adverse health consequences of particular drugs. To this end, MI skills such as “Ask-Tell-Ask” might be deployed (e.g., *Ask* patient what he/she already knows about the effects of crack smoking on the lungs. *Tell* patient how crack might be exacerbating their chronic cough, and *Ask* patient what they think about that information). In this patient-centered spirit, the intervention focuses on the drug of most concern to patients who use multiple drugs.

All brief interventions are audio-recorded and are scored by trained coders using the Motivational Interviewing Treatment Integrity (MITI) instrument [47] to monitor potential drift in the MI protocol as well as to assess for Aim 2 levels of fidelity to MI. While all interventionists are required after training to reach and maintain a specified threshold of MI skill, it is expected there will still be adequate variation in MI skills to allow for an analysis of study Aim 2—testing the degree to which fidelity to the BI model is associated with better outcomes.

It is possible that the intervention may be too brief to have a treatment effect for some patients with severe drug disorders. However, the intervention could still contribute to a referral effect in that patients who participated in a BI may be more likely to seek treatment after being referred. Whether to refer a patient to treatment is determined using the DAST-10 score as a guide in concert with the spirit of the patient-interventionist interaction. After the intervention, a referral to treatment is usually offered if the patient is not overtly hostile to the idea and when the DAST-10 score is 6 or greater in accordance with existing norms [21]. A DAST-10 score less than 3 does not generally trigger a referral. For DAST-10 scores between 3 and 5, the interventionist uses their judgment as to whether to refer based on information obtained from the BI. When in doubt, or whenever the patient asks for help, the interventionists refer. In addition, to preserve internal validity, all individuals assigned to the BI group are given the same referral sheet as individuals assigned to the ECAU group.

A booster session has been shown to enhance the effects of a BI [48] and, for this reason, is included in the present study. The booster session consists of a ten-minute follow-up call approximately one week after the initial intervention. During the call, the interventionist references the plan or option decided upon in the initial intervention and uses MI to follow-up on ambivalence about enacting the plan, generating change talk, or committing to specific goals.

### Training the interventionists

Interventionist training was designed to support the provision of consistently high quality BIs across clinics, between interventionists, and within interventionists over time. It consists of an 8 to 16 hour BI workshop supplemented by one hour of individual supervision once a week for the following three to five weeks. During the individualized supervision, trainees receive tailored coaching with the MI trainer (CD) and feedback on their audio-taped role-plays with standardized patients using their MITI scores. The training protocol was designed to be an enhanced version of manualized trainings recognized for their effectiveness that have been conducted both nationally and internationally [18,44,45,49-53].

### Screening and assessment

It is important to be able to identify individuals with problem drug use reliably and validly, and to be able to do so without interfering with day-to-day medical clinic operations. A brief screening procedure was devised to assess individuals for eligibility in the study; it involves the patient completing a form which includes eligibility criteria and two screening questions: (i) to identify any illicit drug use in the past three months, and (ii) to identify any non-prescribed medication use in the past three months. The screening questions are based on a single-item screening test found to reliably identify drug use in a primary care population [24]. Screen-eligible individuals interested in enrolling in the study are then consented and assessed in a private area on-site with a modified battery (see below) and those randomized to the BI group are provided a BI at the earliest possible time, usually the same day.

### Assessment battery and table

The DAST-10 was selected as an instrument to assess substance abuse severity because of its widespread use over the last decade (including the SAMHSA SBIRT trial in Washington State), its known psychometric properties, the fact that it is self-administered and takes less than 5 minutes to complete, and because it provides a range of scores that have known convergent validity with substance abuse severity defined by other clinical means [21]. This latter characteristic is crucial in stratifying the sample for drug abuse severity prior to randomization.

In order to be consistent with the majority of extant BI studies on alcohol and drug abuse, we use a 12-month assessment window and conduct assessments at baseline, 3, 6, 9, and 12 months. Research assistants conduct follow-up interviews and are blinded to a participant’s intervention versus control group status. Assessments are carried out in-person whenever possible, with phone interview as a backup option. Vigorous outreach is used to complete assessments. The assessments are brief to minimize any unintended intervention effects and to allow the BI to be conducted as close as possible to the screening procedure although we recognize that in-person assessments could potentially contribute to an unintended effect favoring the comparison group. Assessment measures and the times they are administered are summarized in Table 1.

**Table 1:** Assessment measures

**Measure**	**Baseline**	**3 months**	**6 months**	**9 months**	**12 months**
Preliminary Informed consent + 1-item screen	X				
Full informed consent	X				
HIPAA Authorization	X				
Consent for Follow-up Procedures	X				
Release of Information	X				
DAST-10	X				
Thoughts about Drug Use	X	X	X	X	X
Addiction Severity Index (ASI) -Lite	X	X	X	X	X
Treatment Services Review (TSR)	X	X	X	X	X
HIV Risk-Taking Behavior Scale	X	X	X	X	X
EQ-5D-3L	X	X	X	X	X
Urine Sample	X	X	X	X	X

We supplement self-report data collected from participants with data from several administrative sources. These are summarized in Table 2. For the administrative sources, the follow-up time ranges from a minimum of 12 months to a maximum of 24 months, depending on data availability during the time of the study. Obtaining data for a minimum of 12 months will facilitate estimates of public health effects, given prior results of functional effects for substance abuse interventions [13,53,54].

**Table 2:** Administrative data sources

**Data source**	**Owners of data**	**Description of data records**	**Derived measures**
Treatment and Assessment Report Generation Tool (TARGET)	Washington State Department of Social and Health Services (DSHS), Division of Behavioral Health and Recovery (DBHR)	Record of participation in publicly-funded chemical dependency treatment or detoxification services	- Number of days with outpatient treatment visit
			- Number of days of inpatient stays
			- Number of detoxification admissions
Medical records	Medical Center Site of Study	Health care encounters by date, diagnoses	- Number of inpatient hospital stays/days
			- Number of emergency department visits
			- Number of outpatient visits
			- HIV diagnosis date
			- Hepatitis diagnosis date
Billing records	Medical Center Site of Study	Cost of health care encounters by date	Cost of
			-Inpatient hospital stays
			- Emergency department visits
			- Outpatient visits-
Comprehensive Hospital Abstract Reporting System (CHARS)	Washington State Department of Health (DOH)	Hospital discharge data, length of stay, up to 17 diagnoses, demographic information	- Number of inpatient hospital stays/days
Washington State Patrol Arrest Records	Washington State Patrol	All arrests in Washington State including arrest date, nature of offense, level of offense	- Number and type of felony arrests
			- Number and type of gross misdemeanor arrests
Washington State Death Records	Washington State DOH Vital Records	Information about decedent (e.g., age, date of birth, race), place of death, cause-of-death codes, injury information	- Date of death
			- Cause of death

### Choosing the primary outcome variables

Outcome variables were selected specific to each of the study’s aims:

#### Aim 1

For the first aim—to examine whether BI is effective at improving drug use, psychiatric outcomes, select psychosocial outcomes, and attendance in drug abuse treatment—it is important to reliably capture changes in drug use as well as in other variables in as brief a period of time as possible. Because of its widespread use, brevity, and reliability, the validated short form of the Addiction Severity Index (ASI), the ASI-Lite [55], was selected for this purpose along with the Treatment Services Review (TSR) [56]. To further minimize time burden on participants, we only administer items required to compute the ASI composite scores (half the total items) [55].

In order to identify changes in illicit drug use, we use items from the ASI drug section to measure days of any drug use. A urine toxicology screen is used to supplement participants’ responses. In addition, we use the ASI composite scores as comprehensive measures of participants’ medical, psychiatric, legal, family/social, and employment status. The HIV Risk-Taking Behavior Scale is used to assess risk-taking behavior among intravenous drug users [57] and the EQ-5D-3L is used to measure health status [58]. Administrative data are used to supplement ASI data on legal (e.g., arrests) effects. Changes in attendance in drug treatment are obtained from the TSR as well as from state administrative chemical dependency treatment records.

#### Aim 2

The second aim—to test whether fidelity to the BI model is associated with better outcomes—requires a reliable way to assess fidelity. Because of is reliability, cost effectiveness, and brevity, the MITI, which provides a standardized way to rate session audiotapes for degree of MI content, was selected for this purpose [47]. Outcomes for this aim include changes in illicit drug use and drug treatment attendance as measured in the first aim.

#### Aim 3

For the third aim—to estimate the impact of BI on select public health outcomes that are directly related to the hazardous effects of illicit drug use—it is important to identify objective measures reflective of the consequences of illicit drug use. Because of their established tie to illicit drug use, we selected changes in utilization of health care services (including ED visits, inpatient hospitalizations, and hospital days), arrests, and death, as captured in state administrative records [59,60].

#### Aim 4

The fourth aim—to estimate the cost of the intervention, potential cost offsets, and its incremental cost effectiveness —requires valid cost data. The cost of the intervention includes the cost of personnel, materials, and locations for start-up and implementation activities required to screen for drug use and provide a BI. Potential cost offsets will be examined for emergency room visits, outpatient visits, and hospital admissions. The cost measure for all of these medical services includes the direct and indirect costs of providing medical care from the provider perspective. If indicated by study results, we will also assess cost offsets generated by reductions in arrests. If the BI is found to reduce drug use, we will further estimate the incremental cost of the BI intervention compared to its incremental effect on drug use.

### Data analysis

Analyses will compare outcomes for the BI and ECAU groups following the intent-to-treat principle. If indicated, these models will take into account baseline differences between the two groups. Time trend models will be used to examine temporal effects, specifying time of assessment as a categorical variable (baseline, 3, 6, 9, and 12 months). Because assessments are nested within patients and patients are nested within clinics and interventionists, analyses will be conducted with linear mixed models. Clinics will be treated as fixed effects, as they were purposively sampled. In contrast, interventionists were assigned to patients based on availability when a BI had to be administered. For interventionists, random effects will therefore be specified and tested.

#### Power analysis

Power analyses were conducted with PASS software version 08.0.5 [61]. For the primary outcome of drug use reduction, we computed power for the last point in time when data are collected from participants. We expect to enroll 1,000 study participants, 500 each per BI and ECAU group. Assuming an overall response rate of 75%, we would have data from 375 individuals per group at 12 months. According to results from the WASBIRT study, the frequency distribution of number of days drugs were used in the past 30 days follows a Poisson distribution. For individuals who received a BI in WASBIRT, at baseline, average days of drug use in the past 30 days were 3.8 for moderate risk users, 7.0 for high risk users who received only a BI, and 8.7 for high risk users who received a BI and brief therapy and/or chemical dependency treatment. Based on these averages, the study would have 89% power to detect as low as a 2.5% reduction in average drug use days for moderate risk users, at a two-sided alpha level of .05, and an R^2^ between BI indicator variable and other covariates of .25. If the R^2^ between the BI indicator variable and other covariates were 0.5 (a conservative assumption), the power to detect as low as a 2.5% reduction in average drug use days for moderate risk users is 74%; the power to detect a 5% reduction in average drug use days is 99%. For high risk users, the power to detect a reduction in drug use of at least 2.5% is higher. The end-status analysis assumed for these power calculations is considerably conservative, as data from earlier points in time are not used. Thus, the proposed study has sufficient power to address the first two specific aims.

## Discussion

The present study was designed to evaluate the impact of a BI on patients with problem drug use in a primary care setting within a safety net system of care and, in so doing, to be among the few randomized controlled trials of BI for drug abuse in the general medical setting for non-treatment seekers. In addition to capturing post-intervention changes in drug use, psychiatric outcomes, and other psychosocial variables, it also quantifies the impact of BI on the cost of health care, public safety, and mortality in order to inform policy and program planning relative to problem drug use at the local, state, and national levels. As such, it will serve as a policy-relevant trial with broad external validity

## Abbreviations

ASI: Addiction Severity Index; BI: Brief intervention; DAST-10: Drug Abuse Screening Test; ECAU: Enhanced care as usual; ED: Emergency department; HIV: Human immunodeficiency virus; MI: Motivational interviewing; MITI: Motivational Interviewing Treatment Integrity Instrument; PASS: Power analysis and sample size tool; SAMHSA: Substance Abuse and Mental Health Services Administration; SBIRT: Screening, brief intervention and referral to treatment; TSR: Treatment Services Review; WASBIRT: Washington State Screening, Brief Intervention and Referral to Treatment.

## Competing interests

The authors assert that they have no financial or nonfinancial competing interests.

## Authors’ contributions

AK contributed to the design of the study and drafted the manuscript. JMJ is responsible for the statistical design of the study, for data preparation and analyses, and for writing the data analysis section of the manuscript. CD is responsible for the design of the intervention, for training interventionists, and for making substantial contributions to the specific nature of the intervention and the training the interventionist sections of the manuscript. DD contributed to the overall design of the study. KB is responsible for managing day-to-day operations of the project and contributed substantially to the screening and assessment section of the manuscript. SPL participated in training interventionists, in overseeing coding of the taped interviews, and in contributing to sections of the manuscript dealing with the specific nature of the intervention and training the interventionists. RR contributed to the overall design of the study. PRB was responsible for conceiving the study, its design, and for writing the original grant proposal; for making substantial contributions to every aspect of the project’s implementation; and for substantial and critical review of the manuscript for intellectual content. All authors read, provided comments, and approved the final manuscript.
